# Evolving perceptions of point-of-care-technologies: Results from a nationwide survey of healthcare professionals

**DOI:** 10.1017/cts.2025.10149

**Published:** 2025-09-09

**Authors:** Trevor Vigeant, Reilly Riddell, Bernard Ofosuhene, Grace White, Matheus Montenario, Ziyue Wang, Taylor Orwig, Heaven Y. Tatere, Bryan Buchholz, Denise Dunlap, David D. McManus, Ayorkor Gaba, Nathaniel Hafer

**Affiliations:** 1 Department of Medicine, UMass Chan Medical School, Worcester, MA, USA; 2 UMass Center for Clinical and Translational Science, UMass Chan Medical School, Worcester, MA, USA; 3 Program in Molecular Medicine, UMass Chan Medical School, Worcester, MA, USA; 4 Department of Population and Quantitative Health Sciences, UMass Chan Medical School, Worcester, MA, USA; 5 Department of Biomedical Engineering, UMass Lowell, Lowell, MA, USA; 6 Manning School of Business, UMass Lowell, Lowell, MA, USA; 7 Department of Counseling & Clinical Psychology, Teachers College, Columbia University, New York, NY, USA

**Keywords:** Point-of-care technology, needs assessment, cardiology, pulmonology, hematology, sleep medicine

## Abstract

**Background/Objective:**

Point-of-care technologies (POCTs) have grown increasingly prevalent in clinical and at-home settings, offering various rapid diagnostic capabilities. This study presents findings from a nationwide survey conducted between November 2023 and January 2024, capturing clinician perceptions of POCTs.

**Methods:**

The survey was distributed via email to healthcare professionals through academic and industry listservs and through LinkedIn posts. A total of 159 responses were analyzed.

**Results:**

Core priorities, including accuracy, ease of use, and availability, remain consistently valued over the years. However, several perceived benefits, including continuous patient monitoring, diagnostic certainty, and patient management exhibited significant declines in agreement compared to previous years. Despite this, clinician perceptions of POCTs’ abilities to enhance patient–provider communication remained stable. Evolving concerns may reflect heightened expectations and greater scrutiny as these technologies become commonplace. Agreement that POCTs may undermine clinical expertise increases, while concerns related to reimbursement and usability decline. Pilot questions related to artificial intelligence (AI) and machine learning (ML) indicated moderate openness to adopting AI-enhanced POCTs, particularly with tools offering novel clinical insights.

**Conclusions:**

While POCTs continue to be an asset in clinical settings, the findings of this study suggest a shift in provider attitudes toward a more neutral standpoint. Limitations include a low response rate, self-selection, and missing demographic data from a subset of participants. Future surveys will further integrate AI/ML-related questions while prioritizing broader demographic and geographic reach.

## Introduction

Point-of-care technology (POCT) consists of tests conducted in clinical or at home environments for a range of applications, including blood glucose monitoring, home pregnancy testing, and over-the-counter COVID-19 testing [1]. POCTs have significantly evolved in recent years, playing an increasingly vital role in modern healthcare by enabling rapid diagnostics and enhancing patient management. POCTs are also valuable in acute and hyperacute clinical environments, including sepsis and stroke care, and have increasingly been used in research environments to enable real-time data collection and decentralized clinical trials [2–6]. Adopting these technologies has transformed clinical workflows by reducing turnaround times for results, facilitating immediate decision-making, and improving accessibility to critical diagnostic tools [7].

The COVID-19 pandemic served as a catalyst for widespread POCT implementation, emphasizing its importance in mitigating healthcare burdens, particularly in remote and resource-limited settings [8–10]. Between October 2021 and June 2022, 10.7 million self-administered COVID-19 tests were voluntarily reported by users of four manufacturers’ products. In the same period, 361.9 million laboratory-based and point-of-care test results were reported [11]. In the years following the pandemic, the focus has shifted from urgent deployment to refining and optimizing POCT integration within healthcare systems. Advancements aim to establish POCT as an indispensable component of modern healthcare, particularly in preventive medicine and chronic disease management [11]. Moreover, POCTs have expanded beyond infectious diseases to manage other noninfectious and chronic conditions including kidney disease, cancer, diabetes, and certain cardiovascular conditions [7,8,11].

This manuscript describes the results of a clinician-facing survey designed to measure clinician attitudes towards POCT use in clinical settings, adoption patterns, and perceived benefits and limitations over time. The survey was conducted from November 2023 to January 2024. Our analysis builds on previous surveys conducted between 2019 and 2021, which consistently highlighted three priorities of POCTs among healthcare professionals: accuracy, ease of use, and availability [1,12]. Despite technological advancements, these priorities have remained unchanged, underscoring their continued relevance in guiding POCT innovation and adoption. By leveraging survey data collected over multiple years, we aim to assess trends in clinician attitudes, identify persistent barriers to implementation, and provide actionable insights for improving POCT integration. This survey aims to further investigate ongoing concerns while exploring new dimensions of POCT integration, including pilot questions pertaining to perspectives on artificial intelligence (AI) and machine learning (ML) applications.

## Materials and methods

### Survey Development and Distribution

The 2023 POCT survey was a cross-sectional study developed following methodologies established in previous POCT surveys conducted in 2019, 2020 and 2021 [1,8,13]. It was developed to assess healthcare professionals’ perceptions of POCTs, including their importance, benefits, concerns, and clinical integration as their use-cases expand beyond traditional applications. The survey was distributed nationally to diverse healthcare professionals and researchers, ensuring a broad representation of clinical perspectives.

The distribution list included both internal and external email directories, academic medical centers (AMCs), and professional organizations. Contacts were obtained from large directories including the University of Massachusetts Center for Clinical Translational Science, Massachusetts Medical Device Development Center, UMass Memorial Health, Consortia for Improving Medicine with Innovation and Technology, Center for Advancing Point of Care Technologies, National Center for Complementary and Integrative Health (NCCIH), and other directories within the National Heart, Lung, and Blood Institute including the NIH Center for Accelerated Innovations, Small Business Research Initiative, and Research Evaluation and Commercialization Health lists. The broad distribution to academic and clinical professionals likely introduced bias toward regions such as the Northeast and professionals who practice at AMCs. No weighting adjustments were applied to account for regional or specialty-based sampling imbalances.

The survey was launched on 16 November 2023 and closed on 31 January 2024. It was distributed through email and via a LinkedIn invitation post. The exact number of individuals who received the email is unknown; however, based on the listserv populations, it is estimated to be over 15,000. To encourage response, reminder emails were sent monthly for the duration of the survey’s availability. To compensate respondents for their time, a $25 gift card was offered. Although self-exclusion was the only formal exclusion criteria, survey instructions specified the target population as healthcare professionals. While unique links were not utilized, obvious duplicate responses were culled and removed from data analysis. No formal glossary was provided within the survey tool. This may have led to variable interpretations of terms such as “device footprint” and “Clinical Laboratory Improvement Amendments-CLIA” among respondents. 159 responses were eligible for analysis. While all participants were offered compensation for their participation in this survey, 22 respondents chose to provide contact information and were compensated.

The study was deemed exempt from review by the Institutional Review Board (IRB) by the UMass Chan Medical School IRB (Docket #H00018195). We followed the Consensus-Based Checklist for Reporting of Survey Studies to guide the development and reporting of our methodology and results, ensuring transparency and rigor in our process [14].

### Survey Content, Data Collection, and Storage

The first POCT survey was developed in 2019 to assess healthcare professionals’ opinions of POCTs, which specifically compared the responses from providers in cardiovascular medicine to other healthcare professionals. The 2020 survey included additional questions to assess the impact of COVID-19 on healthcare provider impressions of POCT. In 2021, the COVID-19 section was removed and several new questions were added to encapsulate specific issues that arose due to the COVID-19 pandemic. In 2023, additional questions were added to assess the recent rise in AI and ML in POCTs. AI questions were developed based on emerging themes in the digital health literature, internal expert input, and relevance to clinical adoption trends.

Due to the omission or addition of specific questions over various iterations, statistical analyses were used only on questions that were identical between the surveys. The complete survey instrument can be found in the S6 Survey. Survey questions were derived from literature review, prior survey distribution and feedback, and expert consensus. The validity of the survey instrument was supported by its foundation on previously published surveys, expert input, and pilot testing.

The survey contained multiple sections, including provider and patient perceptions of POCTs, perceived benefits and concerns, and the most important characteristics of a POCT. Additional closed-ended questions allowed respondents to list up to five conditions that could benefit from POCT, and demographic information including gender, age, race, ethnicity, profession, practice environment, and years in practice. Likewise, geographic area of practice was collected via a closed-ended question, allowing participants to choose the state in which their practice is located. Participants were not required to answer the questions in totality, and all responses remained anonymous. Missing responses were permitted and not imputed in the data analysis. Questions measuring general POCT matters were adapted from the National Heart, Lung, and Blood Institute’s (NHLBI) 2016 Strategic Vision and a survey developed by the researchers from the Point-of-Care Technology Research Network (POCTRN) center at Johns Hopkins University [4,15]. Questions surrounding business adoption practices of POCTs were adapted from two seminal studies on adopting new technologies [16,17]. Although it was not formally validated through psychometric methods, the survey was iteratively improved based on prior implementations, which included pilot testing. Focus groups were not conducted, but future studies may benefit from such to enhance content validity.

The survey was generated by a Research Electronic Data Capture (REDCap) interface; the secure server is hosted on the UMass Chan network [18]. All data received from participants were transmitted directly into the server for storage and was accessible only by authorized members of the study team.

### Data Analysis

Responses from most questions used a 5-point Likert scale ranging from strongly disagree to strongly agree with the given statement. Survey responses were grouped into two categories for analysis: agreement (responses indicating “Strongly Agree” or “Agree”) and disagreement (responses indicating “Strongly Disagree” or “Disagree”). Checkbox selections for medical specialties were categorized based on an adapted list of standard medical specialties [19].

The importance of various characteristics of POCTs was gathered through a ranking tool. While the ranking tool was adapted from prior surveys, the fixed list of characteristics may limit comprehensiveness. The ordered list may introduce primacy and recency bias. Future iterations may incorporate additional terms including precision, safety, patient comfort, speed of testing, and an optional space for unique responses. Analysis of important characteristics of POCTs was determined using the following system: 1^st^ most important = 3 points, 2^nd^ most important = 2 points, and 3rd most important = 1 point.

Data analysis was conducted using Excel v2502. Chi-square tests were used to assess differences in responses regarding the benefits and concerns of POCTs. Two-proportion *Z*-tests were used to identify significant differences in agree proportions between 2021 and 2023. Heat maps were created to visualize the trends in perception of POCTs over the last 4 survey iterations.

### Results

A total of 159 respondents replied to the 2023 survey. Of those who completed the demographics questions, 49 (48%) identified as male and 51 (46%) as female. Fourteen (13%) respondents were Hispanic or Latino, while 79 (76%) were not. 68 (64%) of the participants self-identified as White, whereas 12 (11%) as Black or African American, 14 (13%) as Asian, 5 (5%) as American Indian or Native Alaskan, and 7 (7%) preferred not to answer. One-quarter of the respondents have been in practice for over 20 years, while over half have been in practice for between 0 and 10 years (25 (24%) for 0 – 5 years and 28 (27%) for 6 – 10 years, respectively). Fifty-one (47%) respondents were physicians, 32 (20%) were advanced practice providers, 14 (13%) were registered nurses, and 12 (11%) identified as “other,” including researchers. Half of the respondents (53) were from the Northeast, while 22 (21%) were from the South, 19 (18%) from the West, and 11 (10%) from the Midwest. Three-quarters, 79 (76%) were employed in hospital or ambulatory clinics (Table 1). Respondents were not required to qualify their answer to either the profession or practice environment “Other” categories; therefore, it is uncertain what specific roles or settings may be represented in the included responses.

**Table 1.** Demographics of survey respondents from the 2023 POCT survey

Participant demographics	Number of respondents (%)
**Gender**	
Male	49 (48)
Female	51 (46)
Undisclosed	4 (4)
**Ethnicity**	
Hispanic or Latino	14 (13)
Not Hispanic or Latino	79 (76)
Prefer Not to Answer	11 (11)
**Race**	
White	68 (64)
Black or African American	12 (11)
Asian	14 (13)
American Indian or Native Alaskan	5 (5)
Prefer not to answer	7 (7)
**Years in practice**	
0 – 5 years	25 (24)
6 – 10 years	28 (27)
11 – 15 years	17 (16)
16 – 20 years	9 (9)
Over 20 years	26 (25)
**Profession**	
Physician (MD/DO)	51 (47)
Advanced practice providers (NP/ APN/ PA)	32 (29)
RN-Registered nurse	14 (13)
Other (Researchers, etc.)	12 (11)
**Geographic region of practice**	
Northeast	53 (50)
South	22 (21)
West	19 (18)
Midwest	11 (10)
Other	1 (1)
**Patient practice environment**	
In-hospital	30 (29)
Ambulatory Clinic	49 (47)
In-home	7 (7)
ER	9 (9)
Other	10 (10)

Demographic characteristics of survey respondents including gender, ethnicity, race, years in practice, profession, and practice environment. Percentages reflect the proportion of total respondents who answered each question.

Respondents were asked to identify the first, second, and third most important characteristics of POCTs when incorporating them into their current practice (Table 2). In 2023, the three most important characteristics were (1) accuracy, (2) ease of use, and (3) availability. These results are consistent with those of 2020 and 2021, while “does not disturb workflow” was the 3^rd^ most important characteristic in 2019 [1,8]. The least important characteristics (rank) in 2023 were (11) Device Footprint, (T9) Sample Type, and (T9) CLIA (Clinical Laboratory Improvement Amendments)-Waived Status. Ruggedness was not included in the 2023 survey.

**Table 2.** Survey values* of the important characteristics of POCT from years 2019 to 2023

Characteristic	2019 (*n* = 154) Weighted points / Rank	2020 (*n* = 287) Weighted points / Rank	2021 (*n* = 168) Weighted points / Rank	2023 (*n* = 106) Weighted points / Rank
Accuracy	325 / 1	651 / 1	386 / 1	235 / 1
Ease of use	175 / 2	345 / 2	195 / 2	132 / 2
Availability	90 / 4	177 / 3	141 / 3	111 / 3
Cost	89 / 5	150 / 4	80 / 4	23 / 6
Reimbursement for testing	36 / 6	85 / 6	40 / 5	21 / 7
Does not disturb workflow	93 / 3	139 / 5	24 / 6	44 / 4
CLIA-waived status	4 / 10	31 / 10	23 / 7	12 / T9
Sample collection	18 / 9	32 / 9	21 / 8	25 / 5
I.S. Connectivity	21 / 8	44 / 7	15 / 9	12 / T9
Sample type	29 / 7	35 / 8	14 / 10	20 / 8
Ruggedness	4 / 11	6 / 11	9 / 11	N/A
Device footprint	1 / 12	1 / 12	2 / 12	9 / 11

*Note:* Ranked importance of POCT characteristics. *Values were determined using a 3-point system (3 points for most important, 2 points for 2^nd^ most important, and 1 point for 3^rd^ most important). Results show comparative longitudinal trends in characteristic prioritization. IS = Information Systems.

## Descriptive Statistics

### POCTs Benefit and Concern Agreement Comparison to 2021

The agreement rates on various statements regarding POCTs between the 2021 and 2023 responses were compared using a two proportion *Z*-test (Table 3). For the perceived benefit statements, four of the fifteen showed a statistically significant decrease (*h* ≥ 0.20 and *p* < 0.05) in agreement from 2021 to 2023. Agreement with the statements “POCTs improve patient management” (*Z* = 3.19, *p* = <0.01, *h* = 0.21), “POCTs improve clinician confidence in decision-making” (*Z* = 3.30, *p* = <0.01, *h* = 0.22), “POCTs decrease overprescribing of drugs such as antibiotics” (*Z* = 3.44, *p* = <0.01, *h* = 0.25), and “POCTs increase diagnostic certainty” (*Z* = 2.78, *p* = <0.01, *h* = 0.20) significantly decreased in 2023 compared to 2021. The most substantial effect sizes observed were for statements related to reimbursement for the cost of the POCT (*h* = 0.30) and overprescription of drugs (*h* = 0.25).

**Table 3.** Significant *Z*-score results for benefit and concern Statements 2021 vs. 2023

Percent agreement benefit	2021	2023	*Z*-score	*p*-value	Cohen’s H
POCTs improve patient management	93.8	81.1	3.19	< 0.01	0.21
POCTs improve clinician confidence in decision making	91.9	77.8	3.30	< 0.01	0.22
POCTs decrease overprescribing of drugs such as antibiotics	76.9	57.0	3.44	< 0.01	0.25
POCTs increase diagnostic certainty	78.8	63.2	2.78	< 0.01	0.20
I might not be reimbursed for the cost of the POCT	36.7	23.1	2.33	0.02	0.30

*Note:* Statistically significant results from two-proportion *Z*-tests highlight significant changes in agreement levels between 2021 and 2023 responses. Cohen’s H indicates effect size. *p* < 0.05 considered significant.

Analysis of the agreement rates regarding perceived concerns yielded one statistically significant decrease in the statement “I might not be reimbursed for the cost of the POCT” (*p* = 0.02, *Z* = 2.33, *h* = 0.30). Notable non-significant decreases included the statements “I can’t provide the necessary quality control for the devices” (*Z* = 1.88, *p* = 0.06, *h* = 0.25) and “The results of POCTs are difficult to interpret/not definitive” (*Z* = 1.90, *p* = 0.06, *h* = 0.25). At the same time, there was a notable non-significant increase for the statement “POCTs undermine clinical expertise” (*Z* = −1.63, *p* = 0.10, *h* = −0.20).

### POCT expected and achieved measurements for the 2023 survey

A Chi-Square test for independence was conducted to examine whether response distributions differed significantly across the years between the categories of agree, neutral, and disagree categories for multiple benefit and concern statements (S1 Table). The highest contributions amongst the benefits statements included diagnostic certainty (*x^2^* = 18.50), patient management (*x^2^* = 9.34), and patient engagement and satisfaction (*x^2^* = 7.52). The highest contributions amongst the concerns statements included the difficulty of interpreting test results (*x^2^* = 26.18), cost concerns (*x^2^* = 20.39), and over-reliance on POCTs (*x^2^* = 16.34). The lone non-significant statement was “POCTs improve patient engagement and buy-in satisfaction” (*p* = 0.08).

### Heat Map Analysis

**Figure 1.**
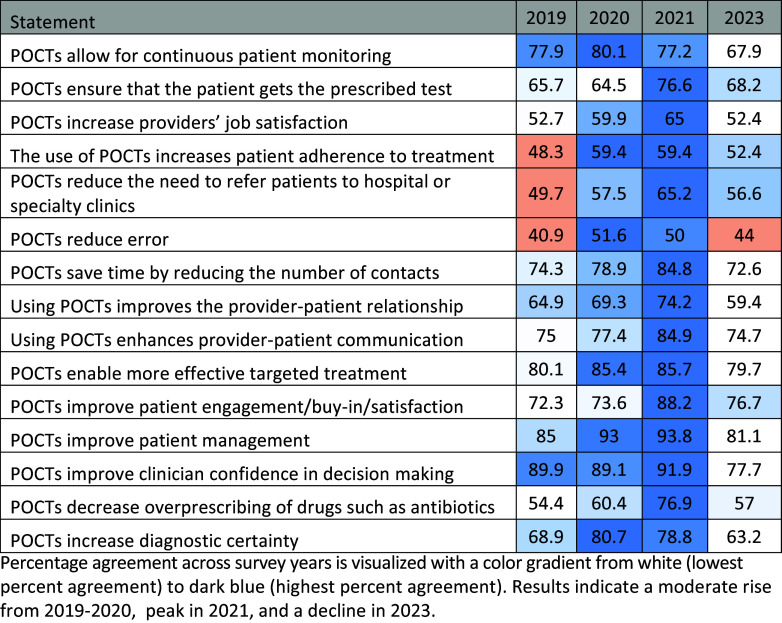
Heat map of agreement with perceived POCT benefits (2019 – 2023).

Heat maps were developed to visualize the trends in agreement of statements of benefit and concern. Figure 1 illustrates the longitudinal responses to the statements of benefit. The color gradient, ranging from white (lowest perceived benefit) to dark blue (highest perceived benefit), demonstrates the year-over-year trends in perceived benefits. Statements with less than fifty percent agreement were shaded orange. The median of each individual statement was set as the mean of the percentage agreement of the aggregate 2019 – 2021 responses.

Percentage agreement across survey years is visualized with a color gradient from white (lowest percent agreement) to dark blue (highest percent agreement). Results indicate a moderate rise from 2019 – 2020, peak in 2021, and a decline in 2023.

**Figure 2.**
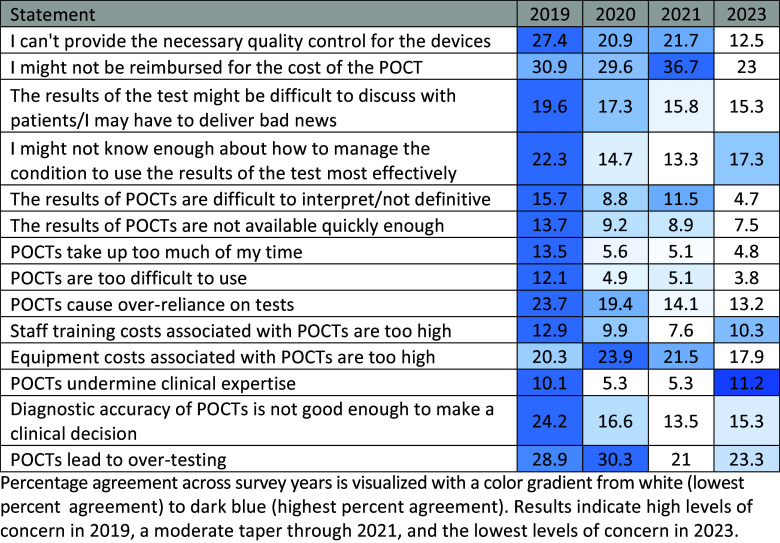
Heat map of agreement with perceived POCT concerns (2019 – 2023).

Figure 2 illustrates responses to the statements of concern. The color gradient, ranging from white (lowest perceived concern) to dark blue (highest perceived concern), demonstrates the year-over-year trends in perceived concerns. The median of each individual statement was set as the mean of the percentage agreement of the aggregate 2019 – 2021 responses.

Percentage agreement across survey years is visualized with a color gradient from white (lowest percent agreement) to dark blue (highest percent agreement). Results indicate high levels of concern in 2019, a moderate taper through 2021, and the lowest levels of concern in 2023.

### Pilot Questions: Artificial Intelligence and Machine Learning

Three pilot questions surrounding the use of AI and ML were introduced in the 2023 study. Thirty-two percent (*n* = 35) of respondents agreed and 19% (*n* = 20) disagreed with the statement “I am likely to adopt a POCT if it utilizes AI/ML.” Fifty-one percent (*n* = 55) of respondents agreed and 6% (*n* = 7) disagreed with the statement “Use of AI/ML in POCT testing can provide novel information that is not currently available,” while 46% (*n* = 49) agreed and 9% (*n* = 10) disagreed with the statement “My practice is eager to adopt AI/ML learning powered innovations in healthcare.” (Table 4)

**Table 4.** Pilot artificial intelligence and machine learning questions

Statement	Agree (%)	Neutral (%)	Disagree (%)
I am more likely to adopt a POCT if it utilizes artificial intelligence/machine learning	35 (32)	53 (49)	20 (19)
Use of artificial intelligence/machine learning in POCT testing can provide novel information that is not currently available	55 (51)	46 (43)	7 (6)
My practice is eager to adopt artificial intelligence/machine learning powered innovations in healthcare.	49 (46)	48 (45)	10 (9)

*Note:* Percent agreement, neutrality, and disagreement with statements assessing clinician attitudes toward pilot AI/ML-enhanced POCT questions.

## Discussion

The 2023 clinician-facing POCT survey highlights significant shifts in healthcare providers’ perceptions and opinions about POCT usage, implementation, and efficacy. The findings demonstrate a tempered perspective, characterized by a reduction in both perceived concerns and perceived benefits. Notably, there was a decline in agreement with several benefit-related statements, indicating reduced confidence in the clinical utility of POCTs. Decreased agreement with statements including “POCTs allow for continuous patient monitoring” and “POCTs improve patient management” may reflect a growing skepticism about the role of POCTs in clinical workflows. This shift in perception could suggest that, with more experience, providers are encountering limitations, such as inconsistent or inaccurate patient use, which may undermine the clinical effectiveness of continuous patient monitoring and management. A mix of benefits and limitations was observed in a survey of clinicians who conduct cervical cancer screening in Indiana [20]. In this study, benefits of POC testing included increased screening, rapid results, and follow up in the same visit, while limitations centered around user errors and POC test accuracy. Similarly, gaining experience with tests and how they impact clinical management were important factors determining POC usage in a cohort of English clinicians [21]. These results may also reflect broader trends in healthcare technology and rising expectations, prompting clinicians to question the real-time reliability and long-term value of these tools.

Perceived benefits peaked in 2021 according to the heat map, with a substantial decrease in the subsequent 2023 survey. Perceived concerns have experienced a more gradual decline throughout the years 2020 – 2023. One notable statement of concern is “POCTs undermine clinical expertise,” which more than doubled in agreement rate (5.3% in 2020 and 2021 to 11.2% in 2023). In conjunction with the statistically significant decreases in agreement with the statements “POCTs improve clinician confidence in decision-making” and “POCTs decrease overprescribing of drugs such as antibiotics,” the results suggest that POCTs are now viewed with more caution. Possible explanations may include providers growing more critical of POCTs as they continue to integrate and interact with them in routine care workflows, or a heightened awareness of their limitations in clinical settings as their weaknesses become more apparent [22]. This may be particularly acute where POCTs are integrated with AI and ML, which can be viewed as a “black box” and susceptible to bias and errors depending on the underlying algorithms and training sets used [23].

However, it is important to note that not all areas of POCT perception have declined. Healthcare providers continue to value POCTs’ interpersonal aspects, as evidenced by non-significant changes related to improving the provider-patient relationship and enhancing communication. The stability in perceived interpersonal benefits demonstrates the continued relevance of POCTs in enhancing patient engagement and satisfaction, a critical aspect of healthcare delivery.

Concerns about POCTs have evolved. Several concerns including “POCTs take up too much of my time” and “POCTs are difficult to use” showed non-significant changes, while concerns about the POCTs undermining clinical expertise have increased. These shifts in concerns may be indicative of a growing complexity related to integrating POCTs into clinical workflows as they are more widely used, along with the challenge of accurate results and maintaining clinical autonomy when necessary.

The decrease in concerns related to the reimbursement for POCTs may suggest that clinicians are facing fewer financial obstacles to POCT implementation. This contrasts with the high levels of concern regarding reimbursement in the prior 2021 survey [1]. This change could indicate that clinicians are either more familiar with reimbursement policies or that financial barriers to POCT adoption have decreased with time.

While our study highlights important trends surrounding the perceived benefits and concerns of POCTs, some limitations must be considered. The sample size was relatively small for the 2023 survey compared to the aggregate data from previous years, which may impact the robustness of our results. Additionally, 49 participants did not complete the demographic questions, limiting our ability to analyze responses by provider characteristics. The study’s low response rate may introduce selection bias, specifically with underrepresentation of non-academic, rural, and non-Northeastern-based providers. Such factors may limit the generalizability of results to other geographic locations and practice settings. Respondents’ academic affiliations may also skew perspectives toward early adopters of technology. Survey respondents practiced in a variety of in-hospital, outpatient, home, and emergency department clinics, suggesting that these findings are generalizable to a number of clinical environments. Additional limitations are described above in the methods section, such as self-selection, multiple participation, and individual variability in survey term interpretation. In future surveys, these questions should be required while offering a “prefer not to answer” option to respect participant privacy while balancing data completeness. Additional qualitative research may uncover the underlying factors influencing these shifting attitudes. Building off the successful AI/ML pilot questions, our future survey will focus on AI/ML technologies, perceptions, integration practices, and trust. Patient perspectives are also critical for the successful implementation of POCTs. While this survey focused on clinician perspectives, our team has previously reported on patient perspectives in two related studies. [24,25] We aim to reach a more diverse healthcare population and expand the settings surveyed to strengthen the generalizability of these findings. Future surveys will prioritize targeted distribution to increase respondent diversity by expanding outreach to providers in rural, community-based, and non-academic settings. To improve geographic representation, partnerships with regional healthcare organizations, rural health associations and professional societies will be prioritized. We will also further explore the use of POCTs for acute and chronic care, and how POCT affects clinical research.

## Conclusion

While POCTs continue to be an asset in clinical settings, the findings of this study suggest a shift in provider attitudes toward a more neutral standpoint – fewer perceived benefits and fewer perceived concerns when compared to three previous surveys between 2019 – 2021. As the healthcare ecosystem continues to evolve, it will be crucial to address these concerns and explore strategies to bolster confidence in POCTs while keeping patient safety and improved outcomes at the forefront. The three most important characteristics of POCTs have remained consistent throughout the 2020, 2021, and 2023 surveys with accuracy, ease of use, and availability being most highly valued. As POCTs and AI/ML-bolstered technologies continue to infiltrate the healthcare ecosystem and clinical workflows, it is crucial to understand clinician perspectives of such tools continuously. Future surveys should include additional AI and ML-centered questions, acknowledging the recent boom in technological advancements, and targeting more diverse and geographically widespread regions to gain a more comprehensive understanding of clinicians’ viewpoints.

## Supporting information

10.1017/cts.2025.10149.sm001 Vigeant et al. supplementary material Vigeant et al. supplementary material

## Supplementary material

The supplementary material for this article can be found at https://doi.org/10.1017/cts.2025.10149.
