# Supplementary material for: Evolving perceptions of point-of-care-technologies: Results from a nationwide survey of healthcare professionals
Source: J Clin Transl Sci. 2025 Sep 9;9(1):e207. doi: 10.1017/cts.2025.10149 (PMC12485566; doi:10.1017/cts.2025.10149)
Supplement: Vigeant et al. supplementary material [file S2059866125101490sup001.pdf]

S1. Chi-Square Analysis of Benefit and Concern Statements Assessing Expected Response Levels. Ex denotes expected response outcome for each statement. DOF = degrees of freedom. P-value <0.05 considered significant.

Statement	Ex Agree (2019-2021)	Ex Neutral (2019-2021)	Ex Dis (2019-2021)	Ex Agree 2022	Ex Neutral 2022	Ex Dis 2022	Chi-Square Contribution	p-value	DOF
POCTs allow for continuous patient monitoring	443.28	101.32	30.40	81.72	18.68	5.60	15.18	<0.01	2
POCTs ensure that the patient gets the prescribed test	391.10	142.45	40.46	72.90	26.55	7.54	7.44	0.02	2
POCTs increase providers' job satisfaction	343.58	201.41	44.01	62.42	36.59	7.99	11.31	<0.01	2
The use of POCTs increases patient adherence to treatment	330.23	223.27	36.50	58.77	39.73	6.50	9.55	<0.01	2
POCTs reduce the need to refer patients to hospital or specialty clinics	337.12	179.57	70.30	60.88	32.43	12.70	8.30	0.02	2
POCTs reduce error	283.87	249.12	61.01	51.13	44.88	10.99	12.76	<0.01	2
POCTs save time by reducing the number of contacts	462.96	99.21	28.83	83.04	17.79	5.17	11.34	<0.01	2
Using POCTs improves the provider-patient relationship	400.96	160.22	28.82	72.04	28.78	5.18	9.89	<0.01	2
Using POCTs enhances provider-patient communication	461.33	104.96	23.70	83.67	19.04	4.30	7.52	0.02	2
POCTs enable more effective targeted treatment	490.84	73.50	23.66	90.16	13.50	4.34	6.16	0.05	2
POCTs improve patient engagement/buy-in/satisfaction	457.46	112.67	22.87	82.54	20.33	4.13	4.86	0.09	2
POCTs improve patient management	529.95	37.31	23.74	95.05	6.69	4.26	19.23	<0.01	2
POCTs improve clinician confidence in decision making	520.69	43.95	25.36	95.31	8.05	4.64	25.35	<0.01	2
POCTs decrease overprescribing of drugs such as antibiotics	368.22	167.60	54.18	66.78	30.40	9.82	10.13	<0.01	2
POCTs increase diagnostic certainty	445.39	104.35	43.27	79.61	18.65	7.73	18.50	<0.01	2
I can't provide the necessary quality control for the devices	123.99	217.41	244.59	22.01	38.59	43.41	19.88	<0.01	2
I might not be reimbursed for the cost of the POCT	180.28	278.92	131.81	31.72	49.08	23.19	16.10	<0.01	2
The results of the test might be difficult to discuss with patients/I may have to deliver bad news	101.02	122.24	366.73	17.98	21.76	65.27	24.25	<0.01	2
I might not know enough about how to manage the condition to use the results of the test most effectively	96.94	150.51	343.55	17.06	26.49	60.45	20.59	<0.01	2
The results of POCTs are difficult to interpret/not definitive	60.17	144.07	384.76	10.83	25.93	69.24	28.18	<0.01	2
The results of POCTs are not available quickly enough	57.58	111.78	416.64	10.42	20.22	75.36	19.16	<0.01	2
POCTs take up too much of my time	41.61	105.29	444.10	7.39	18.71	78.90	26.07	<0.01	2
POCTs are too difficult to use	37.32	101.78	452.91	6.68	18.22	81.09	21.78	<0.01	2
POCTs cause over-reliance on tests	98.28	144.03	345.68	17.72	25.97	62.32	21.35	<0.01	2
Staff training costs associated with POCTs are too high	59.29	192.28	335.43	10.71	34.72	60.57	18.09	<0.01	2
Equipment costs associated with POCTs are too high	128.85	245.83	215.32	23.15	44.17	38.68	16.30	<0.01	2
POCTs undermine clinical expertise	43.18	77.90	469.92	7.82	14.10	85.08	38.13	<0.01	2
Diagnostic accuracy of POCTs is not good enough to make a clinical	101.84	150.22	336.94	18.16	26.78	60.06	15.97	<0.01	2
POCTs lead to over-testing	158.29	198.08	233.63	28.71	35.92	42.37	15.93	<0.01	2

S2. Percent Agreement Benefit 2023 vs. 2019-2021. Two-proportion Z-tests assessing changes in agreement rates between aggregate 2019-2021 data and 2023 responses to perceived benefit statements. Cohen's H indicates effect size. p<0.05 considered significant.

Percent Agreement Benefit	2019-2021	2023	Z-score	p-value	Cohen's H
POCTs allow for continuous patient monitoring	78.78%	67.92%	2.064	0.039	0.247
POCTs ensure that the patient gets the prescribed test	68.12%	68.22%	-0.018	0.985	-0.002
POCTs increase providers' job satisfaction	59.42%	52.34%	1.157	0.247	0.143
The use of POCTs increases patient adherence to treatment	56.61%	52.38%	0.683	0.495	0.085
POCTs reduce the need to refer patients to hospital or specialty clinics	57.58%	56.60%	0.159	0.874	0.020
POCTs reduce error	48.48%	43.93%	0.737	0.461	0.091
POCTs save time by reducing the number of contacts	79.36%	72.64%	1.310	0.190	0.158
Using POCTs improves the provider-patient relationship	69.49%	59.43%	1.732	0.083	0.211
Using POCTs enhances provider-patient communication	78.81%	74.77%	0.790	0.430	0.096
POCTs enable more effective targeted treatment	84.18%	79.63%	0.990	0.322	0.119
POCTs improve patient engagement/buy-in/satisfaction	77.23%	76.64%	0.115	0.908	0.014
POCTs improve patient management	91.20%	81.13%	2.660	0.008	0.296
POCTs improve clinician confidence in decision making	90.17%	77.78%	3.108	0.002	0.344
POCTs decrease overprescribing of drugs such as antibiotics	63.39%	57.01%	1.061	0.289	0.130
POCTs increase diagnostic certainty	77.23%	63.21%	2.610	0.009	0.309

S3. Percent Agreement Concern 2023 vs. 2019-2021. Two-proportion Z-tests assessing changes in agreement rates between aggregate 2019-2021 data and 2023 responses to perceived concern statements. Cohen's H indicates effect size. $p<0.05$ considered significant.

Percent Agreement Concern	2019-2021	2023	Z-score	p-value	Cohen's H
I can't provide the necessary quality control for the devices	22.70%	12.50%	1.993	0.046	0.270
I might not be reimbursed for the cost of the POCT	31.81%	23.08%	1.517	0.129	0.196
The results of the test might be difficult to discuss with patients/I may have to deliver bad news	17.46%	15.24%	0.472	0.637	0.060
I might not know enough about how to manage the condition to use the results of the test most effectively	16.24%	17.31%	-0.230	0.818	-0.028
The results of POCTs are difficult to interpret/not definitive	11.21%	4.72%	1.721	0.085	0.245
The results of POCTs are not available quickly enough	10.24%	7.55%	0.725	0.468	0.095
POCTs take up too much of my time	7.45%	4.76%	0.841	0.400	0.113
POCTs are too difficult to use	6.76%	3.77%	0.987	0.324	0.135
POCTs cause over-reliance on tests	17.35%	13.21%	0.891	0.373	0.115
Staff training costs associated with POCTs are too high	10.05%	10.38%	-0.087	0.931	-0.011
Equipment costs associated with POCTs are too high	22.54%	17.92%	0.898	0.369	0.115
POCTs undermine clinical expertise	6.60%	11.21%	-1.429	0.153	-0.163
Diagnostic accuracy of POCTs is not good enough to make a clinical decision	17.66%	15.24%	0.512	0.608	0.065
POCTs lead to over-testing	27.46%	23.36%	0.744	0.457	0.094

S4. Percent Agreement Benefit 2023 vs. 2021. Two-proportion Z-tests assessing changes in agreement rates between 2021 and 2023 responses to perceived benefit statements. Cohen's H indicates effect size. $p<0.05$ considered significant.

Percent Agreement Benefit	2021	2023	Z-score	p-value	Cohen's H
POCTs allow for continuous patient monitoring	77.22%	67.92%	1.6764	0.0937	0.2089
POCTs ensure that the patient gets the prescribed test	76.58%	68.22%	1.5074	0.1317	0.1875
POCTs increase providers' job satisfaction	64.97%	52.34%	2.0555	0.0398	0.2573
The use of POCTs increases patient adherence to treatment	59.38%	52.38%	1.1236	0.2612	0.1410
POCTs reduce the need to refer patients to hospital or specialty clinics	65.19%	56.60%	1.4071	0.1594	0.1762
POCTs reduce error	50.00%	43.93%	0.9740	0.3300	0.1218
POCTs save time by reducing the number of contacts	84.91%	72.64%	2.4452	0.0145	0.3028
Using POCTs improves the provider-patient relationship	74.21%	59.43%	2.5332	0.0113	0.3157
Using POCTs enhances provider-patient communication	84.91%	74.77%	2.0598	0.0394	0.2545
POCTs enable more effective targeted treatment	85.71%	79.63%	1.3107	0.1900	0.1613
POCTs improve patient engagement/buy-in/satisfaction	88.20%	76.64%	2.5027	0.0123	0.3077
POCTs improve patient management	93.75%	81.13%	3.1851	0.0014	0.3933
POCTs improve clinician confidence in decision making	91.93%	77.78%	3.3025	0.0010	0.4055
POCTs decrease overprescribing of drugs such as antibiotics	76.88%	57.01%	3.4369	0.0006	0.4268
POCTs increase diagnostic certainty	78.75%	63.21%	2.7812	0.0054	0.3453

S5. Percent Agreement Concern 2023 vs. 2021. Two-proportion Z-tests assessing changes in agreement rates between 2021 and 2023 responses to perceived concern statements. Cohen's H indicates effect size. $p<0.05$ considered significant.

Percent Agreement Concern	2021	2023	Z-score	p-value	Cohen's H
I can't provide the necessary quality control for the devices	21.66%	12.50%	1.8847	0.0595	0.2453
I might not be reimbursed for the cost of the POCT	36.71%	23.08%	2.3281	0.0199	0.2996
The results of the test might be difficult to discuss with patients/I may have to deliver bad news	15.82%	15.24%	0.1280	0.8981	0.0161
I might not know enough about how to manage the condition to use the results of the test most effectively	13.29%	17.31%	-0.8936	0.3715	-0.1118
The results of POCTs are difficult to interpret/not definitive	11.46%	4.72%	1.9001	0.0574	0.2530
The results of POCTs are not available quickly enough	8.92%	7.55%	0.3936	0.6939	0.0499
POCTs take up too much of my time	5.06%	4.76%	0.1104	0.9121	0.0139
POCTs are too difficult to use	5.06%	3.77%	0.4932	0.6219	0.0629
POCTs cause over-reliance on tests	14.01%	13.21%	0.1863	0.8522	0.0235
Staff training costs associated with POCTs are too high	7.59%	10.38%	-0.7858	0.4320	-0.0976
Equipment costs associated with POCTs are too high	21.52%	17.92%	0.7147	0.4748	0.0904
POCTs undermine clinical expertise	5.70%	11.21%	-1.6318	0.1027	-0.2010
Diagnostic accuracy of POCTs is not good enough to make a clinical decision	13.46%	15.24%	-0.4035	0.6866	-0.0507
POCTs lead to over-testing	21.02%	23.36%	-0.4519	0.6514	-0.0565

S6. Point of Care Testing Survey 2023

Welcome!

Thank you for taking the time to complete our survey. We want your expert opinion about what qualities of point of care technologies (POCTs) are most important to health care providers. Moreover, we would like to better understand the decision-making process of implementing these newer technologies into everyday practice. Our hope is that theresults of this survey will identify areas of need and encourage new research and development. Your input will have a direct impact on the technology and use cases we focus on and fund. In this study we are interested in point of care technologies for heart, lung, blood, and sleep diseases.

Point of Care Tests (POCTs) are defined as tests that can be done onsite (in the clinic, ER, home, hospital) with results available during the visit and before the patient leaves. We are asking about POCTs on samples or measurements taken from the body, including blood, urine, and other bodily fluids.

This survey should take about 10 minutes. Participation is voluntary and you can stop at any time. Your responses are anonymous. Compensation will be provided for completing the survey ($25).

If you have any questions please email Nate Hafer, PhD at nathaniel.hafer@umassmed.edu

---

This question was placed here only so that REDCap would recognize a section break. It is not relevant to the study and is hidden to prevent responses. ________________________________

Point of Care Tests (POCTs) are defined as tests that can be done onsite (in the clinic, ER, home, hospital) with results available during the visit and before the patient leaves. We are asking about POCTs on samples or measurements taken from the body, including blood, urine, and other bodily fluids. Examples of POCT include blood glucose, blood pressure, EKG, and carbon monoxide breath tests.

POCT in the clinic could improve how you care for patients.	◯ strongly agree<br>◯ agree<br>◯ neutral<br>◯ disagree<br>◯ strongly disagree
POCT at home could improve how you care for patients.	◯ strongly agree<br>◯ agree<br>◯ neutral<br>◯ disagree<br>◯ strongly disagree
Do you believe that the use of POCT has changed since the start of the COVID-19 pandemic?	◯ greatly increased<br>◯ somewhat increased<br>◯ no change<br>◯ somewhat decreased<br>◯ greatly decreased
To what extent do your patients understand the purpose and benefits of POCT?	◯ not at all<br>◯ not very well<br>◯ somewhat well<br>◯ moderately well<br>◯ very well
Do you believe that POCT improves patient outcomes?	◯ strongly agree<br>◯ agree<br>◯ neutral<br>◯ disagree<br>◯ strongly disagree
How often do you receive training on using POCT effectively and safely?	◯ very frequently<br>◯ frequently<br>◯ occasionally<br>◯ rarely<br>◯ never
Do you believe that POCT has affected health equity? (Health equity is the achievement of the highest level of care for all)	◯ Yes<br>◯ No
Has POCT reduced or increased health inequities?	◯ greatly reduced<br>◯ somewhat reduced<br>◯ no difference<br>◯ somewhat increased<br>◯ greatly increased

Please characterize your opinion on the following prompts regarding the use of Artificial Intelligence/Machine Learning:

	strongly disagree	disagree	neutral/not sure	agree	strongly agree
I am more likely to adopt a POCT if it utilizes Artificial Intelligence/Machine Learning	○	○	○	○	○
Use of Artificial Intelligence/Machine Learning in POCT testing can provide novel information that is not currently available	○	○	○	○	○
My practice is eager to adopt AI/ML powered innovations in healthcare	○	○	○	○	○

Select up to 5 conditions for which a POCT could help you make a DIAGNOSIS of a disease. Please select the conditions whether POCTs currently exist for the condition or not.

- ☐ Heart Diseases (including hypertension)
- ☐ Lung Diseases
- ☐ Blood Diseases
- ☐ Allergies
- ☐ Skin Diseases
- ☐ Eye Diseases
- ☐ Ear Nose and Throat Diseases
- ☐ Cancer
- ☐ Stomach and Intestinal Diseases
- ☐ Thyroid and Endocrine Diseases
- ☐ Infections including COVID
- ☐ Diseases of metabolism
- ☐ Neurological Diseases like stroke
- ☐ Psychiatric Diseases like depression
- ☐ Birth and Gynecological Diseases
- ☐ Bone and muscle Diseases (not joints)
- ☐ Joint Diseases like arthritis
- ☐ Kidney Diseases
- ☐ Bladder Diseases

Select up to 5 conditions for which a point of care test could help you MONITOR or MANAGE disease. Please select the conditions whether the POCTs currently exist for the condition or not.

- ☐ Heart Diseases (including hypertension)
- ☐ Lung Diseases
- ☐ Blood Diseases
- ☐ Allergies
- ☐ Skin Diseases
- ☐ Eye Diseases
- ☐ Ear Nose and Throat Diseases
- ☐ Cancer
- ☐ Stomach and Intestinal Diseases
- ☐ Thyroid and Endocrine Diseases
- ☐ Infections including COVID
- ☐ Diseases of metabolism
- ☐ Neurological Diseases like stroke
- ☐ Psychiatric Diseases like depression
- ☐ Birth and Gynecological Diseases
- ☐ Bone and muscle Diseases (not joints)
- ☐ Joint Diseases like arthritis
- ☐ Kidney Diseases
- ☐ Bladder Diseases

We are listing potential BENEFITS of POCTs. What is your perception about the following statements?

	strongly disagree	disagree	neutral / not sure	agree	strongly agree
a. POCTs increase diagnostic certainty	○	○	○	○	○
b. POCTs decrease overprescribing of drugs such as antibiotics	○	○	○	○	○
c. POCTs improve clinician confidence in decision making	○	○	○	○	○
d. POCTs improve patient management	○	○	○	○	○
e. POCTs improve patient engagement/buy-in/satisfaction	○	○	○	○	○
f. POCTs enable more effective targeted treatment	○	○	○	○	○
g. Using POCTs enhances provider-patient communication	○	○	○	○	○
h. Using POCTs improves the provider-patient relationship	○	○	○	○	○
i. POCTs save time by reducing the number of contacts (repeat visits, telephone conversations, etc.)	○	○	○	○	○
j. POCTs reduce error	○	○	○	○	○
k. POCTs reduce the need to refer patients to hospital or specialty clinics	○	○	○	○	○
l. The use of POCTs increases patient adherence to treatment	○	○	○	○	○
m. POCTs increase providers' job satisfaction	○	○	○	○	○
n. POCTs ensure that the patient gets the prescribed test	○	○	○	○	○
o. POCTs allow for continuous patient monitoring	○	○	○	○	○
p. Faster turnaround time with POCT test results increases the opportunity for immediate feedback by a health care provider	○	○	○	○	○
q. An advantage of a POCT is a decreased need for additional patient travel to a blood collection site for central lab testing	○	○	○	○	○

r. POCT fingerstick blood test results can be as clinically useful as test results from a venous blood draw sent to a central lab	○	○	○	○	○
s. Environmental hygiene and bloodborne pathogen exposure during specimen collection and handling for POCT can be as safe as that of venous blood draw procedures for central laboratory testing	○	○	○	○	○

We are listing potential CONCERNS of POCTs. What is your perception of the following statements?

	strongly disagree	disagree	neutral / not sure	agree	strongly agree
a. POCTs lead to over-testing	○	○	○	○	○
b. Diagnostic accuracy of POCTs is not good enough to make a clinical decision	○	○	○	○	○
c. POCTs undermine clinical expertise	○	○	○	○	○
d. Equipment costs associated with POCTs are too high	○	○	○	○	○
e. Staff training costs associated with POCTs are too high	○	○	○	○	○
f. POCTs cause over-reliance on tests	○	○	○	○	○
g. POCTs are too difficult to use	○	○	○	○	○
h. POCTs take up too much of my time	○	○	○	○	○
i. The results of POCTs are not available quickly enough	○	○	○	○	○
j. The results of POCTs are difficult to interpret/not definitive	○	○	○	○	○
k.The results of the test might be difficult to discuss with patients/I may have to deliver bad news	○	○	○	○	○
l. I might not know enough about how to manage the condition to use the results of the test most effectively	○	○	○	○	○

m. I might not be reimbursed for the cost of the POCT	○	○	○	○	○
n. I can't provide the necessary quality control for the devices	○	○	○	○	○
o. I am concerned about the accuracy of some commercial POCT that my patients use	○	○	○	○	○

What characteristic of a point of care technology is 1st, 2nd, and 3rd most important when incorporating it into your regular practice?

	1st most important	2nd most important	3rd most important
1. availability	○	○	○
2. ease of use	○	○	○
3. accuracy	○	○	○
4. sample type	○	○	○
5. sample collection	○	○	○
6. does not disrupt workflow	○	○	○
7. cost	○	○	○
8. device footprint	○	○	○
9. reimbursement for testing	○	○	○
10. information systems connectivity	○	○	○
11. CLIA-waived status	○	○	○

The next set of questions asks that you reflect on the strategic decision-making styles of your practice and/or the hospital or health care setting where you spend the most time.
Rate the extent to which you agree with the following statements characterizing the external business environment within which your practice operates.

	strongly disagree	disagree	neutral / not sure	agree	strongly agree
My practice has growing investment opportunities.	○	○	○	○	○

Rate the extent to which you agree with the following characterizations regarding the philosophy of top leaders within your practice.

	strongly disagree	disagree	neutral / not sure	agree	strongly agree
Communication channels are open and encouraged.	○	○	○	○	○
There is a strong emphasis on updating our practices to meet new challenges.	○	○	○	○	○

The leaders of our practice are willing to take bold measures to achieve our organization's mission.	○	○	○	○	○

Rate the extent to which you agree with the following characterizations about your organization's culture.

	strongly disagree	disagree	neutral / not sure	agree	strongly agree
It is generally known throughout our practice that our intention is to grow as big and as fast as possible.	○	○	○	○	○
My practice is committed to addressing future problems that impact society-at-large.	○	○	○	○	○
My practice routinely collaborates with outside organizations in order to adapt to new challenges.	○	○	○	○	○

Please characterize your practice's adoption of new lines of heart, lung, blood, and sleep disease products or services over the past 5 years by indicating your agreement with the following statements:

	strongly disagree	disagree	neutral / not sure	agree	strongly agree
We adopt new products and/or services frequently to meet our patients' growing needs.	○	○	○	○	○
Utilizing new point of care technology is a top priority in our practice.	○	○	○	○	○

Please characterize your practice's relationship with its competitors by rating your agreement with the following statements:

	strongly disagree	disagree	neutral / not sure	agree	strongly agree
My practice often introduces new products and/or services before my competitors do.	○	○	○	○	○
My practice has a good understanding of the products and/or services that our competing institutions offer.	○	○	○	○	○

Please characterize your practice's decision-making tendencies by rating your agreement with the following statements:

	strongly disagree	disagree	neutral / not sure	agree	strongly agree
The leaders of our practice pursue a cautious, 'wait-and-see' position to minimize making costly decisions.	◯	◯	◯	◯	◯

Demographic Questions

What is your gender?

◯ Woman
◯ Man
◯ Transgender
◯ Non-binary
◯ Other (please specify)
◯ Prefer not to respond

please specify 'other'

____

What is your specialty? (select all that apply)

☐ Cardiology
☐ Family or Internal Medicine
☐ Pulmonology
☐ Hematology
☐ Emergency Medicine
☐ Infectious Disease
☐ Sleep Medicine
☐ Other

Please describe other specialty:

____

What is your profession?

◯ MD-Medical Doctor
◯ DO-Doctor of Osteopathy
◯ NP-Nurse Practitioner
◯ APN-Advanced Practice Nurse
◯ PA-Physicians' Assistant
◯ RN-Registered Nurse
◯ Other

Please describe your profession:

____

What state is your practice located in?

- ◯ Alabama
- ◯ Alaska
- ◯ Arizona
- ◯ Arkansas
- ◯ California
- ◯ Colorado
- ◯ Connecticut
- ◯ Delaware
- ◯ District of Columbia
- ◯ Florida
- ◯ Georgia
- ◯ Hawaii
- ◯ Idaho
- ◯ Illinois
- ◯ Indiana
- ◯ Iowa
- ◯ Kansas
- ◯ Kentucky
- ◯ Louisiana
- ◯ Maine
- ◯ Maryland
- ◯ Massachusetts
- ◯ Michigan
- ◯ Minnesota
- ◯ Mississippi
- ◯ Missouri
- ◯ Montana
- ◯ Nebraska
- ◯ Nevada
- ◯ New Hampshire
- ◯ New Jersey
- ◯ New Mexico
- ◯ New York
- ◯ North Carolina
- ◯ North Dakota
- ◯ Ohio
- ◯ Oklahoma
- ◯ Oregon
- ◯ Pennsylvania
- ◯ Rhode Island
- ◯ South Carolina
- ◯ South Dakota
- ◯ Tennessee
- ◯ Texas
- ◯ Utah
- ◯ Vermont
- ◯ Virginia
- ◯ Washington
- ◯ West Virginia
- ◯ Wisconsin
- ◯ Wyoming
- ◯ Other

Please describe other location:

____

What is your practice environment?

- ◯ In-home
- ◯ ambulatory clinic
- ◯ Emergency Room
- ◯ in-hospital
- ◯ other

Please specify other practice environment.	__________________________________
What is the best approximation of your primary ambulatory practice?	◯ Public Health Clinic<br>◯ Federally Qualified Health Center<br>◯ College/University<br>◯ Health Maintenance Organization<br>◯ Private Community Clinic<br>◯ Private-Single Practitioner Setting<br>◯ Private-Multiple Practitioner Setting<br>◯ Hospital-owned Single Group Practice
How many years have you practiced after completing your terminal training/degree?	◯ 0-5 years<br>◯ 6-10 years<br>◯ 11-15 years<br>◯ 16-20 years<br>◯ Over 20 years
What is your ethnicity?	◯ Hispanic or Latino<br>◯ Not Hispanic or Latino<br>◯ I prefer not to respond
What is your race?	☐ White<br>☐ Black or African American<br>☐ Asian<br>☐ American Indian or Alaska Native<br>☐ Native Hawaiian or Other Pacific Islander<br>☐ Other<br>☐ I prefer not to respond
Specify other race	__________________________________
Would you like to be compensated for completing this survey?	◯ Yes<br>◯ No
Please provide your full name and email address, we will reach out and provide details on payment.<br>Thank you!	__________________________________
